# Diagnostic Value of Serum Procalcitonin in Patients with Convulsion in Emergency Department, an Observational Study

**DOI:** 10.3390/antibiotics9100683

**Published:** 2020-10-08

**Authors:** Hisashi Murakami, Hiromu Naraba, Takashi Gondo, Masaki Mochizuki, Hidehiko Nakano, Yuji Takahashi, Tomohiro Sonoo, Hideki Hashimoto, Kensuke Nakamura

**Affiliations:** 1 Department of Emergency and Critical Care Medicine, Hitachi General Hospital, 2-1-1, Jonan-cho, Hitachi, Ibaraki 317-0077, Japan; nrbhrm@gmail.com (H.N.); t.gondo.0222@gmail.com (T.G.); kurakan72@gmail.com (M.M.); be.rann1988jp@gmail.com (H.N.); yuji.mail@icloud.com (Y.T.); sonopy77@gmail.com (T.S.); hidehashimoto-tky@umin.ac.jp (H.H.); mamashockpapashock@yahoo.co.jp (K.N.); 2 TXP Medical Co., Ltd, 7-3-1 Hongo Bunkyo-ku, Tokyo 113-8485, Japan

**Keywords:** convulsion, creatine kinase, epilepsy, meningitis, procalcitonin, seizure

## Abstract

Procalcitonin (PCT), a widely used biomarker for bacterial infections, is sometimes measured in convulsion patients to distinguish bacterial infections including bacterial meningitis. However, serum PCT elevation is reported in several other conditions. This study assessed the diagnostic value of serum PCT concentrations in convulsion patients. This study examined a convulsion group: patients admitted to our critical care center during April 2018 through September 2019 via the emergency department presenting with convulsions. Randomly sampled patients admitted without convulsions were categorized as a non-convulsion group. Serum PCT analysis was performed with consideration of whether or not the patient had an infection. Diagnostic values of serum PCT for bacterial infection were evaluated for convulsion and non-convulsion patients using the positive likelihood ratio of PCT. This study found 84 patients as eligible for the convulsion group; 1:2 matched 168 control patients were selected as non-convulsion group members. The positive likelihood ratio for bacterial infection was found to be significantly lower in the convulsion group than in the control group (1.94 vs. 2.65) when setting the positive cut-off for PCT as 0.5 ng/mL. Convulsion patients had a higher PCT value. The positive likelihood ratio for patients without bacterial infection was lower.

## 1. Introduction

Procalcitonin (PCT), a sepsis-related protein, was first found to be elevated in patients with sepsis in 1993 [1]. Today, it is used widely as a biomarker for bacterial infections. Activated adherent macrocytes and various tissues produce PCT during inflammatory response, and especially during response to bacterial infection [2,3]. In contrast to bacterial infections, viral infections usually induce modest PCT production because interferon-γ inhibits adipocytes from producing PCT [4]. Consequently, it is said that the PCT value can help to distinguish bacterial from viral meningitis [5,6,7].

Distinguishing infectious complications including bacterial meningitis is often necessary for the clinical management of convulsion patients in emergency departments. Although a PCT value would be useful to exclude bacterial meningitis in patients with convulsion presentation, elevation of serum PCT has been reported for several conditions other than bacterial infection. These conditions include severe trauma, surgery [8], burn injury [9], post cardiopulmonary arrest [10], severe liver failure [3], severe pancreatitis [11], and cardiac shock [12]. Any one of them can confuse diagnostic decisions based on a PCT value.

In addition to these clinical conditions, clinical experience at our facility indicates that convulsion itself is often accompanied by elevated PCT. However, the mechanism for this relation remains unclear.

This study analyzed serum PCT concentrations in convulsion patients in an emergency department. It was conducted to clarify the diagnostic value of PCT for bacterial infection in convulsion patients.

## 2. Materials and Methods

### 2.1. Study Design

This is an observational case-control study. It was conducted at a tertiary care center in Japan.

### 2.2. Study Population

**Figure 1:**
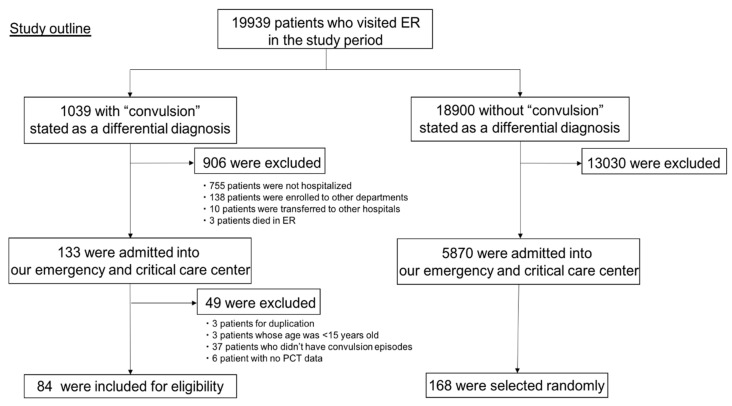
Outline of patient selection.

The study population consisted of convulsion patients and matched control patients, all of whom were admitted to the Emergency and Critical Care Center of Hitachi General Hospital. At our hospital, the PCT value was measured for every patient admitted through the ED. “Convulsion group” patients during April 2018 through September 2019 (18 months) were extracted from our ED database using a search of data related to the chief complaint, diagnosis, and history of present illness using the words “convulsion” and “seizure”. Patients younger than 15 years old and those who had no laboratory test were excluded from the “convulsion group”. Patients who had visited the ED within 7 days prior were also excluded to prevent another infection effect. The “non-convulsion group” included patients who were admitted to the Emergency and Critical Center of Hitachi General Hospital during the same time period and who had no reported seizure-like activity. Patients younger than 15 years old, who had no laboratory data, and who had visited the ED within 7 days prior, were also excluded. Twice as many patients of the convulsion group were selected randomly, based on a random coefficient, as from the “non-convulsion group” (Figure 1).

Each group’s patients were categorized into two groups: “infection” and “no infection”. Patients who did not receive antibiotics within two days following admission were defined as “no infection”. The others were categorized as the “infection” group. In addition, patients who were assessed as having no infection by an infectious disease expert’s chart review but who received antibiotics within two days following admission were categorized into the “no infection” group. Therefore, the “infection group” includes all bacterial infections, irrespective of central nervous system infections.

### 2.3. Data Collection

All data, including age, sex, vital signs, and laboratory data, were collected through ED and an Intensive Care Unit admission database. Vital signs were recorded on arrival to the emergency room (ER). The maximum PCT value of the admission day and the next day were selected as target PCT values. In addition, the following blood test data were collected: white blood cell (WBC), hemoglobin (Hb), platelet (Plt), total protein (TP), albumin (Alb), aspartate transaminase (AST), alanine transaminase (ALT), bilirubin (Bil), blood urea nitrogen (BUN), creatinine (Cre), C-reactive protein (CRP), lactate (Lac), ammonia (NH_3_), CK, and PCT. The degree of consciousness on arrival was evaluated using the Japan coma scale (JCS), with results of four designations: JCS-0, alert; JCS-1, awake with no stimuli; JCS-2, rousable but reverts to the earlier state if stimulus stops; JCS-3, cannot be roused using any forceful stimulus. The causes of convulsion were classified into seven categories by chart review: idiopathic epilepsy, stroke, old cerebral infarction, brain tumor, central nervous system infection, metabolic encephalopathy, and others.

### 2.4. Statistics

Continuous variables showing a normal distribution were presented as the mean with the standard deviation. Other continuous variables were presented as medians with an interquartile range. Categorical variables such as the sex distribution, presence of infection, JCS, and cause of convulsion are presented as the number and percentage of the total study sample.

Results were evaluated by the positive likelihood ratio of PCT for the infection, which is defined as the proportion of “infection group” patients among the patients with a positive PCT value. The positive likelihood ratios of PCT for infections in the convulsion group and the non-convulsion group were compared. The cut-off of PCT was set as 0.5 ng/mL following the standard value of bacterial infection [13,14]: PCT ≥ 0.5 ng/mL is positive; PCT < 0.5 ng/mL is negative. We also evaluated those data when setting the PCT cut-off line as PCT ≥ 1.0 ng/mL and ≥2.0 ng/mL as sensitivity analyses.

Significance was inferred for *p* < 0.05. Statistical analyses were conducted using software (JMP ver. 15; SAS Institute Inc., Tokyo, Japan).

## 3. Results

### 3.1. Bullet Points

- Positive likelihood ratio for bacterial infection is lower in convulsion patients.
- Procalcitonin might be elevated by convulsion itself.

### 3.2. Patients Results

During the study period, 19,939 patients visited the Hitachi General Hospital ED. Of those, 1039 patients presented with some kind of convulsion. 133 patients with convulsion were admitted to the Emergency and Critical Care Center. From them, 49 patients were excluded according to the exclusion criteria explained above. Finally, 84 patients were collected as the “convulsion group”. Regarding the control group, 5870 patients without convulsion were admitted to the Hospital. From them, 1:2 matched 168 patients were sampled randomly as the “non-convulsion group”. Figure 1 shows a patient selection outline.

Table 1 presents the patients’ basic characteristics. The *t*-tests and Wilcoxon tests revealed significant differences in age, infection positive rate, low JCS proportion, blood pressure, heart rate, and PCT between those of the “convulsion group” and “non-convulsion group”. The mean value of PCT and the proportion of patients with positive PCT were lower in the convulsion group than in the non-convulsion group (0.24 ng/mL vs. 0.33 ng/mL, 33% vs. 46%, convulsion vs. control).

Procalcitonin results obtained from the convulsion group and the non-convulsion group are presented in Table 2. When setting the PCT cut-off line as 0.5 ng/mL, the positive likelihood ratio for bacterial infection was significantly lower in the convulsion group than in the non-convulsion group (1.94 vs. 2.65). When setting the PCT cut-off line as PCT > 1.0 ng/mL and >2.0 ng/mL, similar results of the positive likelihood ratio were respectively obtained (2.30 vs. 3.04, 1.71 vs. 3.15) (Table 2).

## 4. Discussion

This study assessed the diagnostic value of PCT for bacterial infection in convulsion patients admitted to the ED. Our results demonstrated that the positive likelihood ratio of PCT for bacterial infection was significantly lower in convulsion patients, suggesting low effectiveness of PCT as a diagnostic marker to distinguish bacterial infection in convulsion patients.

Several non-infectious conditions described in the background section were reported to trigger the PCT elevation. However, no report describes correlation between convulsions themselves and the PCT value. Because convulsion occurs in 20% of meningitis cases [15], PCT values in convulsion patients in the ED seemed helpful in supporting appropriate decisions about lumbar puncture to rule out bacterial meningitis. Nevertheless, our study results suggest that convulsion itself would trigger an elevation in PCT. Furthermore, the lower positive likelihood ratio for bacterial infection in convulsion patients makes it difficult to interpret the PCT value as an indicator of bacterial meningitis.

Several explanations of the higher PCT values in post-convulsion patients can be considered. Additional analysis showed that the elevation of PCT is associated moderately with CK elevation in both groups. The CK value was significantly higher in the PCT positive patients than in PCT negative patients (*p* = 0.028). This result suggests that destruction of striated muscle following a convulsion is correlated to the elevation of PCT and CK. This hypothesis is compatible with earlier reports describing that rhabdomyolysis is associated with the elevation of PCT [16]. This outcome might be related to elevated cytokines such as interleukin-6, which are predominantly produced inside skeletal muscle during strenuous exercise that stimulates monocytes and various tissues to produce PCT [17,18].

This study has several limitations. First, this study was conducted at a single center. For that reason, the external generalizability of its results is limited. Second, patients who returned home without admission were not included. Third, the diagnosis of convulsion was not based on physiological procedures such as electroencephalography.

## 5. Conclusions

In ED convulsion patients, the serum PCT value was elevated. The positive likelihood ratio of serum PCT for bacterial infection was lower.

## Figures and Tables

**Table 1:** Basic patient characteristics of the two groups. Infection, Japan Coma Scale, systolic blood pressure, heart rate, and procalcitonin were higher in the convulsion group than in the control group.

Group	Convulsion	Non-Convulsion	
*n*	84	168	*p* Value
Age (years)	66.1 ± 18.5	72.2 ± 17.6	0.0094 *
Sex (male), (%)	52 (61)	99 (58)	0.64
SOFA score	4.90 ± 3.2	5.94 ± 3.7	0.075
APACHEII score	17.3 ± 8.9	18.0 ± 9.7	0.68
Infection (%)	21 (25)	77 (45)	<0.001 *
Japan Coma Scale			<0.001 *
0–3 (%)	33 (39)	112 (67)	
10–30 (%)	10 (12)	28 (16)	
100–300 (%)	41 (49)	28 (17)	
Body temperature (°C)	36.7 ± 4.4	36.6 ± 3.6	0.83
Systolic blood pressure (mmHg)	153.8 ± 40.0	133.4 ± 42.9	0.0029 *
Heart rate (bpm)	108.6 ± 30.5	94.0 ± 25.3	0.0008 *
Respiratory rate (cpm)	22.9 ± 6.9	22.2 ± 9.1	0.55
SpO2 (%)	95.8 ± 4.4	94.3 ± 11.9	0.96
Procalcitonin (ng/mL)	0.24 (0.05,1.09)	0.33 (0.08,2.58)	0.028 *
Procalcitonin > 0.5 ng/mL (%)	28 (33)	78 (46)	0.029 *

*: *p* < 0.05. SD, standard deviation; SOFA score, sequential organ failure assessment score; APACHEII score, acute physiology and chronic health evaluation II score.

**Table 2:** Procalcitonin results and positive likelihood ratio for bacterial infection in two groups (convulsion and non-convulsion groups) when setting the three PCT cut-off values.

Group	Convulsion		Non-Convulsion	
Infection	Infection	No Infection	Infection	No Infection
Total	21	63	77	91
PCT cut-off 0.5 ng/mL				
PCT positive	11	17	54	24
PCT negative	10	46	23	67
Positive likelihood ratio	1.94		2.65	
PCT cut-off 1.0 ng/mL				
PCT positive	10	13	49	19
PCT negative	11	50	28	72
Positive likelihood ratio	2.30		3.04	
PCT cut-off 2.0 ng/mL				
PCT positive	8	14	40	15
PCT negative	13	49	37	76
Positive likelihood ratio	1.71		3.15	
